# Rituximab: mechanisms and applications

**DOI:** 10.1054/bjoc.2001.2127

**Published:** 2001-11

**Authors:** P W M Johnson, M J Glennie

**Affiliations:** CRC Medical Oncology Unit, Tenovus Research Laboratory Cancer Sciences Division, Southampton University School of Medicine


*British Journal of Cancer* (2001) **85**(11), 1619–1623
© 2001 Cancer Research Campaign
doi: 10.1054/ bjoc.2001.2127, available online at http://www.idealibrary.com on IDEAL
http://www.bjcancer.com

### Rituximab: mechanisms and applications

**PWM Johnson[1]* and MJ Glennie[2]**

[1] CRC Medical Oncology Unit and [2] Tenovus Research Laboratory Cancer Sciences Division, Southampton University School of Medicine

Rituximab, a chimeric monoclonal antibody (mAb) which targets the CD20 molecule on the B-cell surface, was the first antibody to be licensed for the treatment of malignancy. This represents the culmination of more than two decades of intensive investigation into the use of mAb in lymphoma, and points the way to a new era of cancer-specific therapy. There are many questions still outstanding about the precise cellular effects of the antibody, but emerging clinical data suggest that it may represent a step forward in the treatment of B-cell malignancies. Unfortunately the application of rituximab is running well ahead of the evidence, with 30 000 patients per annum receiving it in the USA at a cost of over $400 million in 2000, a figure which is rising steeply. As many as 80% of newly diagnosed patients with large cell lymphoma are reported to be receiving treatment including the antibody, despite the absence of any fully published evidence of efficacy in a randomised trial. This situation needs to be corrected as rapidly as possible, and it will be important to complete the trials now underway.

### MECHANISMS OF ACTION

It is likely that the action of rituximab against lymphoma is due as much to direct signalling in the malignant B-cells following CD20 ligation as to recruitment of effectors such as complement and antibody-directed cellular cytotoxicity (ADCC) (Vitetta and Uhr, 1994; Cragg et al, 1999; Ghetie et al, 2001). This is important because it suggests that the combination of antibodies with chemo- or radio-therapy may enhance rather than diminish the therapeutic effect.

CD20 was one of the first B-cell-specific markers to be identified in the early investigations of the cell surface phenotype with mAb (Stashenko et al, 1980). Although murine anti-CD20 molecules were available for some time, their clinical application was largely restricted to in vitro purging of bone marrow for transplantation, and later work with radioconjugates. The principal limitations to their use in vivo were a short half-life, normally less than 20 hours, and the potential for stimulating human anti-mouse antibodies (HAMA), resulting in the formation of circulating immune complexes that were therapeutically inactive and could cause severe adverse effects if generated in sufficient quantity. It was only when molecular techniques allowed the generation of chimeric mAb through replacement of the mouse constant domains with human ones that the half life was extended to several days and serial treatments were not impeded by HAMA responses.

The CD20 molecule is a 33–37 kDa phosphoprotein with a predicted structure comprising 4 transmembrane regions, a 44 amino acid extracellular loop and cytoplasmic N- and C-termini (Einfeld et al, 1988). It was always an attractive target for mAb therapy due to its high expression on almost all normal B cells and B-cell malignancies and its resistance to internalisation or shedding from the plasma membrane following ligation (Press et al, 1987). No natural ligand has been identified for CD20, and progress in understanding the biology of the molecule has been hampered by the inability to generate anti-mouse CD20 mAb. CD20 knockout mice show no definitive phenotype (O'Keefe et al, 1998), but ligation of CD20 with mAb does result in a variety of interesting events, including triggering of intracellular signalling cascades and altered calcium transport. Furthermore, stimulation of B cells with anti-CD20 mAbs and phorbol esters results in enhanced phosphorylation of CD20, again suggesting a role in transmembrane signalling and control of cell growth (Tedder and Schlossman, 1988). Functional studies with a variety of CD20 antibodies have shown modulatory effects on B cell activation, proliferation and differentiation (Golay et al, 1985; Smeland et al, 1985), and activation of protein tyrosine kinases. (Kansas and Tedder, 1991). There is limited evidence that CD20 functions as a calcium channel subunit (reviewed in Tedder and Engel (1994)). This fits with findings that CD20 exists on the cell surface as a homo-oligomer (Bubien et al, 1993), and regulates cell cycle progression possibly through maintaining calcium levels and thus inhibiting progress from $G_1$ to S phase (Kanzaki et al, 1995). Recent data show that ligation of CD20 with mAb leads directly to apoptosis and appears to use the same intracellular pathway(s) as apoptosis triggered via the B-cell receptor (Mathas et al, 2000). This was most clearly demonstrated by the close correlation between the sensitivity of various B-cell lines to anti-CD20- and anti-BCR-mediated apoptosis and the similarities in the proapoptotic proteins, such as BAX, that these reagents up-regulate.

A variety of molecules appear to associate with CD20 in the cell membrane. A complex of CD20, MHC class I and II, and members of the TM4SF has been described (Szollosi et al, 1996), and other studies have shown co-localisation with Src-family kinases Lyn, Fyn and Lck (Deans et al, 1995). There is also evidence to suggest that stimulation with anti-CD20 results in the redistribution of CD20 into membrane rafts (Deans et al, 1998), with CD20 and the Src-family kinases anchored in these (Casey, 1995). This redistribution is accompanied by the appearance of an uncharacterised 50 kDa tyrosine-phosphorylated protein in the same compartment (Polyak et al, 1998). Other B-cell growth-regulating effects seen upon CD20 ligation include activation of phospholipase Cγ, interruption of the IL6/STAT 3 pathway (Alas and Bonavida, 2001) and up-regulation of c-Myc. In addition, recent results have suggested that engaging CD20 can inhibit production of IL10, which may act as an autocrine growth factor as well as influencing the local T-cell repertoire (Alas et al, 2001).

*Correspondence to:* P Johnson

* Professor Johnson has acted as a symposium speaker and advisory board member for Roche Pharmaceuticals.

The effects of CD20 ligation have been studied mainly in transformed B-cell lines, and little work has been carried out in isolated primary cells from human lymphoma or in vivo. Rituximab is unusually effective in both ADCC and complement-mediated cytotoxicity, and in some cell lines at least, induces potent apoptosis when sufficiently crosslinked (Shan et al, 1998; Mathas et al, 2000). While the relative importance of these processes is not known, Ravetch and colleagues have recently used immunoglobulin constant region receptor (FcR)-deficient mice to show an important role for both stimulatory and inhibitory FcR in mediating the therapeutic activity of rituximab (Clynes et al, 2000). In a series of elegant experiments this group demonstrated that full therapeutic activity of rituximab and similar anti-cancer mAb was only achieved in mice expressing stimulatory, γ-chain-associated, FcγR. These data were consistent with FcR contributing significantly to the antitumour activity in vivo. However, many mAb which are highly active in ADCC, for example anti-CD19, have failed to produce effects in clinical studies and it is clear that multiple mechanisms are likely to operate in vivo. Interestingly, even in γ-chain deficient mice, which do not express stimulatory FcR and could not mediate ADCC, mAb still retain some therapeutic activity in various models (Clynes et al, 1998). Such therapy could be due to complement-mediated killing, in which rituximab is highly active, particularly against tumour cells lacking or with blocked complement inhibitor molecules, CD55 and CD59 (Golay et al, 2000; Harjunpaa et al, 2000). It is also possible that the direct cytotoxic activity of rituximab is important and that FcR-expressing cells can influence this pathway. By providing a multivalent array of FcR, even low-affinity receptors will be able to hyper-crosslink Ab-coated target cells. Shan and co-workers (Shan et al, 1998) have shown that FcR-expressing effector cells, which increase crosslinking, can enhance apoptosis induced by anti-CD20 mAb bound to B-cell targets. Likewise, Mathas (Mathas et al, 2000) showed that while highly crosslinked rituximab was very effective in inhibiting the growth of B-cell lines, soluble anti-CD20 had little activity.

In summary, it is evident that rituximab can control lymphoma growth by several mechanisms, including ADCC, complement activation and triggering direct intracellular signalling cascades. The relative contribution of each of these in vivo remains uncertain and is likely to keep immunologists busy for some time to come.

### CLINICAL APPLICATION

The earliest studies tested rituximab alone as a treatment for recurrent CD20$^+$ lymphoma. A dose of 375 mg m$^{-2}$ weekly for 4 weeks was established as the standard. A small randomised trial comparing a higher dose of 500 mg m$^{-2}$ did not show a significant difference in response rates (Coiffier et al, 1998). The 'pivotal' study included 166 patients with lymphoma of Working Formulation grades A–C in whom the disease had recurred despite initially successful treatment, in many cases quite extensive (McLaughlin et al, 1998). There was a response rate of 48%, with a median response duration of 13 months, a result confirmed in other similar studies from Europe. This treatment has now been tested in the whole range of B-cell lymphomas, and the results are summarised in Table 1. There is a modest response rate in diffuse large cell and mantle cell lymphomas, but a rather lower rate in lymphocytic lymphoma/CLL, possibly due to their lower density of CD20 molecules. The approach of either increasing the dose of antibody or prolonging the course of treatment has been tested in this group, with some suggestion of a higher response rate in phase II studies (Byrd et al, 2001; O'Brien et al, 2001). In all disease types the responses are of limited duration, around a median of 1 year.

**Table 1** Responses of recurrent lymphoma to rituximab

| Study (Reference) | Number of patients | % response rate | Median response duration (months) |
|---|---|---|---|
| Pivotal: WF A-D (McLaughlin et al, 1998) | 166 | 48 | 13 |
| UK follicular (Foran et al, 2000b) | 70 | 46 | 11 |
| European mantle cell (Foran et al, 2000a) | 87 | 34 | 12 |
| Small lymphocytic (Foran et al, 2000c) | 28 | 14 | NS |
| Small lymphocytic (dose intense) (Byrd et al, 2001) | 33 | 45 | 10 |
| European large Cell (Coiffier et al, 1998) | 30 | 37 | > 8 |

The early studies confirmed the tolerable side-effect profile of rituximab, with the only common serious events being infusion-related hypotension, fever and rigors, seen almost entirely with the first infusion and not recurring on continued treatment. Patients with large numbers of circulating lymphoma cells are more likely to suffer adverse effects, probably mediated by complement activation. The antibody has a substantial effect upon the numbers of circulating normal B-cells, but these recover after an interval of 3 – 6 months and there is consequently little impact upon immunoglobulin levels. The incidence of opportunistic infection is low.

The moderate side-effect profile encouraged use of the antibody as first-line therapy, and the studies which have been performed in low-grade lymphoma suggest that the response rate may be up to 75% in this setting (Hainsworth et al, 2000; Colombat et al, 2001). This observation may be particularly interesting for the group of patients with low-grade lymphoma for whom conservative 'watchful waiting' would normally be the approach, but in whom prolonged remission might be achieved with brief duration low-toxicity antibody therapy. Trials are in progress to test this. In this context it is interesting that re-treatment with rituximab appears to be well tolerated, without substantially different side effects to those seen with the first course and a very low incidence of antichimeric antibodies (Davis et al, 2000), so that use early in the illness may not compromise later therapy.

One outcome that has been extensively studied with monoclonal antibody treatment is the ability to achieve 'molecular remission', namely the eradication of polymerase chain reaction (PCR)-detectable residual lymphoma cells as indicated by the presence of a characteristic translocation such as the t(14;18) BCL2-IgH rearrangement in follicular lymphoma or t(11;14) BCL1-IgH in mantle cell lymphoma (Foran et al, 2000b; Colombat et al, 2001; Czuczman et al, 2001). These tests are still unproven as surrogate endpoints (Johnson et al, 1999), but a common observation is that rituximab therapy is frequently capable of clearing the peripheral blood and bone marrow of PCR-detectable cells, although lymph node masses may remain. Thus in the French study of initial therapy for follicular lymphoma 17 of 30 (57%) patients went from BCL2-IgH PCR positive to negative in peripheral blood after

 *© 2001 Cancer Research Campaign*

**Table 2** Summary of results in the GELA study

| | R-CHOP (*n* = 169) | CHOP (*n* = 159) | |
|---|---|---|---|
| Progression on therapy (%) | 8 | 21 | |
| Recurrence (%) | 8 | 16 | |
| Complete response (%) | 76 | 60 | $P = 0.004$ |
| Event-free survival at 12 months (%) | 69 | 49 | $P < 0.0005$ |
| Overall survival at 12 months (%) | 83 | 68 | $P < 0.01$ |

50 days, and at 12 months 16 of 26 (62%) remained PCR negative (Colombat et al, 2001). Although PCR is of limited usefulness as a predictive test in this setting, the results do suggest that clearing the blood and bone marrow of disease prior to progenitor cell harvesting may be possible with rituximab, and this approach is being tested in several studies of 'in vivo purging' before high-dose therapy (Magni et al, 2000; Voso et al, 2000).

The demonstration of efficacy as a single agent has led to the investigation of rituximab as a component of combination chemo-immunotherapy. Whilst it might have been thought that co-administration with chemotherapy and corticosteroids would mitigate the effect of the antibody through disruption of immune effectors, in practice these misgivings have not been borne out. Studies in the initial therapy of both follicular and diffuse large cell lymphomas have shown a very high response rate, with up to 60% complete responses (Czuczman et al, 1999; Vose et al, 2001), and the most provocative finding was in the French Groupe d'etudes des lymphomes de l'adulte (GELA) study (Coiffier et al, 2000). This randomised patients over the age of 60 with newly diagnosed diffuse large B-cell lymphoma to conventional chemotherapy (CHOP) or the same treatment together with rituximab in each cycle. With a median follow up of 12 months there were significant differences in response rate, time to progression and overall survival in favour of the CHOP + rituximab arm (Table 2). Almost all the difference between the arms appears to derive from an early improvement in the response rate. On the basis of these results a randomised study examining a similar question (ECOG study 4494) was closed early in the United States, although a large study addressing the question in younger patients continues in Europe (MINT: http://www.kompetenznetz-lymphome.de/mint/). Similar studies are being conducted in low-grade lymphoma, in some cases with second randomisations to maintenance treatment with the antibody versus observation.

The combination of anti-CD20 with irradiation has been extensively investigated using murine antibodies as radioconjugates, but to date few studies have attempted to use chimeric molecules, out of concern for their prolonged half-life in vivo and the risk of high normal tissue doses. A single trial in recurrent low-grade lymphoma has compared rituximab to radioimmunotherapy (RIT) using the parent murine antibody (C2-B8) conjugated to $^{90}$Y, with an apparently higher response rate in the radioimmunotherapy arm (80 versus 56%: $P < 0.002$) but apparently no more durable remissions (Witzig et al, 2000). The difficulty of delivering more than one dose of RIT with murine antibodies owing to HAMA responses may restrict the effectiveness of this strategy.

Apart from the relatively common B-cell lymphomas, rituximab has also been tested in the treatment of a variety of other entities such as myeloma, Waldenstrom's macroglobulinaemia and hairy cell leukaemia (Hoffman and Auerbach, 2000; Zinzani et al, 2000). Case reports and small series have reported responses in all of these, but as yet it has not found a definite place in the routine scheme of management. Post-transplant lymphoproliferative disease (PTLD) has also been treated with rituximab, which is an attractive alternative to cytotoxics in patients who are already immunosuppressed. Several series have been reported, with high response rates particularly among patients who have Epstein–Barr-positive disease (Kuehnle et al, 2000). The largest series of 32 patients treated in France reported a 69% response rate, although the follow up was too short to establish how durable these were likely to be (Milpied et al, 2000).

Finally, the prospect that CD20 targeting might modulate normal B-cell function has led to the testing of rituximab as therapy for a variety of antibody-mediated autoimmune diseases such as idiopathic thrombocytopenic purpura, rheumatoid arthritis and cryoglobulinaemia. There are preliminary reports of efficacy in some cases, with 3 of 9 corticosteroid refractory patients with ITP responding to rituximab in one series (Saleh et al, 2000). Further studies will determine whether or not this is a useful addition to the treatment of autoimmunity.

### CONCLUSION

Rituximab has rapidly found applications in the management of most types of B-cell lymphoma, although at present the evidence base is unsatisfactory. In low-grade disease it has activity as a single agent and there is no doubt that responses are seen in patients for whom other types of treatment have failed, with minimal associated toxicity. It will be extremely difficult to conduct convincing randomised trials in this group of patients when the level of efficacy has been demonstrated so clearly with phase II studies. In more aggressive lymphoma randomised studies are being conducted, at least in Europe, and the results will be critical for determining the future use of the antibody. Better understanding of the mechanisms of action may be most helpful in guiding the use of combination therapy, both with cytotoxics and possibly other types of immunotherapy.

### REFERENCES

Alas S and Bonavida B (2001) Rituximab inactivates signal transducer and activation of transcription 3 (STAT3) activity in B-non-Hodgkin's lymphoma through inhibition of the interleukin 10 autocrine/paracrine loop and results in down-regulation of Bcl-2 and sensitization to cytotoxic drugs. *Cancer Res* **61**: 5137–5144

Alas S, Emmanouilides C and Bonavida B (2001) Inhibition of interleukin 10 by rituximab results in down-regulation of bcl-2 and sensitization of B-cell non-Hodgkin's lymphoma to apoptosis. *Clin Cancer Res* **7**: 709–723

Bubien JK, Zhou LJ, Bell PD, Frizzell RA and Tedder TF (1993) Transfection of the CD20 cell surface molecule into ectopic cell types generates a $Ca^{2+}$ conductance found constitutively in B lymphocytes. *J Cell Biol* **121**: 1121–1132

Byrd JC, Murphy T, Howard RS, Lucas MS, Goodrich A, Park K, Pearson M, Waselenko JK, Ling G, Grever MR, Grillo–Lopez AJ, Rosenberg J, Kunkel L and Flinn IW (2001) Rituximab using a thrice weekly dosing schedule in B-cell chronic lymphocytic leukemia and small lymphocytic lymphoma demonstrates clinical activity and acceptable toxicity. *J Clin Oncol* **19**: 2153–2164

Casey PJ (1995) Protein lipidation in cell signaling. *Science* **268**: 221–225

Clynes R, Takechi Y, Moroi Y, Houghton A and Ravetch JV (1998) Fc receptors are required in passive and active immunity to melanoma. *Proc Natl Acad Sci USA* **95**: 652–656

Clynes RA, Towers TL, Presta LG and Ravetch JV (2000) Inhibitory Fc receptors modulate in vivo cytoxicity against tumor targets. *Nat Med* **6**: 443–446

Coiffier B, Haioun C, Ketterer N, Engert A, Tilly H, Ma D, Johnson PW, Lister TA, Feuring-Buske M, Radford JA, Capdeville R, Diehl V and Reyes F (1998) Rituximab (anti-CD20 monoclonal antibody) for the treatment of patients with relapsing or refractory aggressive lymphoma: A multicenter phase II study. *Blood* **92**: 1927–1932

Coiffier B, Lepage E, Herbrecht R, Tilly H, Solal-Celigny P, Munck JN, Bouabdallah R, Lederlin P, Sebban C, Morel P, Haioun C, Salles G, Molina T and Gisselbrecht C (2000) Mabthera (rituximab) plus CHOP is superior to CHOP alone in elderly patients with diffuse large B-cell lymphoma (DLCL): interim results of a randomized GELA trial. *Blood* **96**: 223a

Colombat P, Salles G, Brousse N, Eftekhari P, Soubeyran P, Delwail V, Deconinck E, Haioun C, Foussard C, Sebban C, Stamatoullas A, Milpied N, Boue F, Taillan B, Lederlin P, Najman A, Thieblemont C, Montestruc F, Mathieu-Boue A, Benzohra A and Solal-Celigny P (2001) Rituximab (anti-CD20 monoclonal antibody) as single first-line therapy for patients with follicular lymphoma with a low tumor burden: clinical and molecular evaluation. *Blood* **97**: 101–106

Cragg MS, French RR and Glennie MJ (1999) Signaling antibodies in cancer therapy. *Current Opinion Immunol* **11**: 541–547

Czuczman MS, Grillo-Lopez AJ, White CA, Saleh M, Gordon L, LoBuglio A, Jonas C, Klipperstein D, Dallaire BK and Varns C (1999) Treatment of patients with low-grade B-cell lymphoma with the combination of chimeric anti-CD20 monoclonal antibody and CHOP chemotherapy. *Journal of Clinical Oncology* **17**: 268–276

Czuczman MS, Grillo-Lopez AJ, McLaughlin P, White CA, Saleh M, Gordon L, LoBuglio AF, Rosenberg J, Alkuzweny B and Maloney D (2001) Clearing of cells bearing the bcl-2 [t(14;18)] translocation from blood and marrow of patients treated with rituximab alone or in combination with CHOP chemotherapy. *Ann Oncol* **12**: 109–114

Davis TA, Grillo-Lopez AJ, White CA, McLaughlin P, Czuczman MS, Link BK, Maloney DG, Weaver RL, Rosenberg J and Levy R (2000) Rituximab anti-CD20 monoclonal antibody therapy in non-Hodgkin's lymphoma: safety and efficacy of retreatment. *J Clin Oncol* **18**: 3135–3143

Deans JP, Kalt L, Ledbetter JA, Schieven GL, Bolen JB and Johnson P (1995) Association of 75/80-kDa phosphoproteins and the tyrosine kinases Lyn, Fyn and Lck with the B cell molecule CD20. Evidence against involvement of the cytoplasmic regions of CD20. *J Biol Chem* **270**: 22632–22638

Deans JP, Robbins SM, Polyak MJ and Savage JA (1998) Rapid redistribution of CD20 to a low density detergent-insoluble membrane compartment. *J Biol Chem* **273**: 344–348

Einfeld DA, Brown JP, Valentine MA, Clark EA and Ledbetter JA (1988) Molecular cloning of the human B cell CD20 receptor predicts a hydrophobic protein with multiple transmembrane domains. *Embo J* **7**: 711–717

Foran JM, Cunningham D, Coiffier B, Solal-Celigny P, Reyes F, Ghielmini M, Johnson PW, Gisselbrecht C, Bradburn M, Matthews J and Lister TA (2000a) Treatment of mantle-cell lymphoma with Rituximab (chimeric monoclonal anti-CD20 antibody): analysis of factors associated with response. *Ann Oncol* **11**: 117–121

Foran JM, Gupta RK, Cunningham D, Popescu RA, Goldstone AH, Sweetenham JW, Pettengell R, Johnson PW, Bessell E, Hancock B, Summers K, Hughes J, Rohatiner AZ and Lister TA (2000b) A UK multicentre phase II study of rituximab (chimaeric anti-CD20 monoclonal antibody) in patients with follicular lymphoma, with PCR monitoring of molecular response. *Br J Haematol* **109**: 81–88

Foran JM, Rohatiner AZ, Cunningham D, Popescu RA, Solal-Celigny P, Ghielmini M, Coiffier B, Johnson PW, Gisselbrecht C, Reyes F, Radford JA, Bessell EM, Souleau B, Benzohra A and Lister TA (2000c) European phase II study of rituximab (chimeric anti-CD20 monoclonal antibody) for patients with newly diagnosed mantle-cell lymphoma and previously treated mantle-cell lymphoma, immunocytoma, and small B-cell lymphocytic lymphoma. *Journal of Clinical Oncology* **18**: 317–324

Ghetie MA, Bright H and Vitetta ES (2001) Homodimers but not monomers of Rituxan (chimeric anti-CD20) induce apoptosis in human B-lymphoma cells and synergize with a chemotherapeutic agent and an immunotoxin. *Blood* **97**: 1392–1398

Golay J, Zaffaroni L, Vaccari T, Lazzari M, Borleri GM, Bernasconi S, Tedesco F, Rambaldi A and Introna M (2000) Biologic response of B lymphoma cells to anti-CD20 monoclonal antibody rituximab in vitro: CD55 and CD59 regulate complement-mediated cell lysis. *Blood* **95**: 3900–3908

Golay JT, Clark EA and Beverley PC (1985) The CD20 (Bp35) antigen is involved in activation of B cells from the G0 to the G1 phase of the cell cycle. *J Immunol* **135**: 3795–3801

Hainsworth JD, Burris HA, 3rd, Morrissey LH, Litchy S, Scullin DC, Jr., Bearden JD, 3rd, Richards P and Greco FA (2000) Rituximab monoclonal antibody as initial systemic therapy for patients with low-grade non-Hodgkin lymphoma. *Blood* **95**: 3052–3056

Harjunpaa A, Junnikkala S and Meri S (2000) Rituximab (anti-CD20) therapy of B-cell lymphomas: direct complement killing is superior to cellular effector mechanisms. *Scand J Immunol* **51**: 634–641

Hoffman M and Auerbach L (2000) Bone marrow remission of hairy cell leukaemia induced by rituximab (anti-CD20 monoclonal antibody) in a patient refractory to cladribine. *Br J Haematol* **109**: 900–901

Johnson PWM, Swinbank K, MacLennan S, Colomer D, Debuire B, Diss T, Gabert J, Gupta RK, Haynes A, Kneba M, Lee MS, Macintyre E, Mensink E, Moos M, Morgan GJ, Neri A, Johnson A, Reato G, Salles G, van't Veer MB, Zehnder JL, Zucca E, Selby PJ and Cotter FE (1999) Variability of polymerase chain reaction detection of the Bcl2-IgH translocation in an International multicentre study. *Ann Oncol* **10**: 1349–1354

Kansas GS and Tedder TF (1991) Transmembrane signals generated through MHC class II, CD19, CD20, CD39, and CD40 antigens induce LFA-1-dependent and independent adhesion in human B cells through a tyrosine kinase-dependent pathway. *J Immunol* **147**: 4094–4102

Kanzaki M, Shibata H, Mogami H and Kojima I (1995) Expression of calcium-permeable cation channel CD20 accelerates progression through the G1 phase in Balb/c 3T3 cells. *J Biol Chem* **270**: 13099–13104

Kuehnle I, Huls MH, Liu Z, Semmelmann M, Krance RA, Brenner MK, Rooney CM and Heslop HE (2000) CD20 monoclonal antibody (rituximab) for therapy of Epstein–Barr virus lymphoma after hemopoietic stem-cell transplantation. *Blood* **95**: 1502–1505

Magni M, Di Nicola M, Devizzi L, Matteucci P, Lombardi F, Gandola L, Ravagnani F, Giardini R, Dastoli G, Tarella C, Pileri A, Bonadonna G and Gianni AM (2000) Successful in vivo purging of CD34-containing peripheral blood harvests in mantle cell and indolent lymphoma: evidence for a role of both chemotherapy and rituximab infusion. *Blood* **96**: 864–869

Mathas S, Rickers A, Bommert K, Dorken B and Mapara MY (2000) Anti-CD20- and B-cell receptor-mediated apoptosis: evidence for shared intracellular signaling pathways. *Cancer Res* **60**: 7170–7176

McLaughlin P, Grillo-Lopez AJ, Link BK, Levy R, Czuczman MS, Williams ME, Heyman MR, Bence-Bruckler I, White CA, Cabanillas F, Jain V, Ho AD, Lister J, Wey K, Shen D and Dallaire Bk (1998) Rituximab chimeric anti-CD20 monoclonal antibody therapy for relapsed indolent lymphoma: half of patients respond to a four-dose treatment program. *Journal of Clinical Oncology* **16**: 2825–2833

Milpied N, Vasseur B, Parquet N, Garnier JL, Antoine C, Quartier P, Carret AS, Bouscary D, Faye A, Bourbigot B, Reguerre Y, Stoppa AM, Bourquard P, Hurault de Ligny B, Dubief F, Mathieu-Boue A and Leblond V (2000) Humanized anti-CD20 monoclonal antibody (Rituximab) in post transplant B-lymphoproliferative disorder: a retrospective analysis on 32 patients. *Ann Oncol* **11**: 113–116

O'Brien SM, Kantarjian H, Thomas DA, Giles FJ, Freireich EJ, Cortes J, Lerner S and Keating MJ (2001) Rituximab dose-escalation trial in chronic lymphocytic leukemia. *J Clin Oncol* **19**: 2165–2170

O'Keefe TL, Williams GT, Davies SL and Neuberger MS (1998) Mice carrying a CD20 gene disruption. *Immunogenetics* **48**: 125–132

Polyak MJ, Tailor SH and Deans JP (1998) Identification of a cytoplasmic region of CD20 required for its redistribution to a detergent-insoluble membrane compartment. *J Immunol* **161**: 3242–3248

Press OW, Appelbaum F, Ledbetter JA, Martin PJ, Zarling J, Kidd P and Thomas ED (1987) Monoclonal antibody 1F5 (anti-CD20) serotherapy of human B cell lymphomas. *Blood* **69**: 584–591

Saleh MN, Gutheil J, Moore M, Bunch PW, Butler J, Kunkel L, Grillo-Lopez AJ and LoBuglio AF (2000) A pilot study of the anti-CD20 monoclonal antibody rituximab in patients with refractory immune thrombocytopenia. *Semin Oncol* **27**: 99–103

Shan D, Ledbetter JA and Press OW (1998) Apoptosis of malignant human B cells by ligation of CD20 with monoclonal antibodies. *Blood* **91**: 1644–1652

Smeland E, Godal T, Ruud E, Beiske K, Funderud S, Clark EA, Pfeifer-Ohlsson S and Ohlsson R (1985) The specific induction of myc protooncogene expression in normal human B cells is not a sufficient event for acquisition of competence to proliferate. *Proc Natl Acad Sci USA* **82**: 6255–6259

Stashenko P, Nadler LM, Hardy R and Schlossman SF (1980) Characterization of a human B lymphocyte-specific antigen. *J Immunol* **125**: 1678–1685

Szollosi J, Horejsi V, Bene L, Angelisova P and Damjanovich S (1996) Supramolecular complexes of MHC class I, MHC class II, CD20, and tetraspan molecules (CD53, CD81, and CD82) at the surface of a B cell line JY. *J Immunol* **157**: 2939–2946

Tedder TF and Schlossman SF (1988) Phosphorylation of the B1 (CD20) molecule by normal and malignant human B lymphocytes. *J Biol Chem* **263**: 10009–10015

 *© 2001 Cancer Research Campaign*

Tedder TF and Engel P (1994) CD20: a regulator of cell-cycle progression of B lymphocytes. *Immunol Today* **15**: 450–454

Vitetta ES and Uhr JW (1994) Monoclonal antibodies as agonists: an expanded role for their use in cancer therapy. *Cancer Res* **54**: 5301–5309

Vose JM, Link BK, Grossbard ML, Czuczman M, Grillo-Lopez A, Gilman P, Lowe A, Kunkel LA and Fisher RI (2001) Phase II study of rituximab in combination with chop chemotherapy in patients with previously untreated, aggressive non-Hodgkin's lymphoma. *J Clin Oncol* **19**: 389–397

Voso MT, Pantel G, Weis M, Schmidt P, Martin S, Moos M, Ho AD, Haas R and Hohaus S (2000) In vivo depletion of B cells using a combination of high-dose cytosine arabinoside/mitoxantrone and rituximab for autografting in patients with non-Hodgkin's lymphoma. *Br J Haematol* **109**: 729–735

Witzig TE, White CA, Gordon li, Murray JI, Wiseman GA, Emmanouilides C, Czuczman MS, Shen D, Multani P and Grillo-López AJ (2000) Final results of a randomized controlled study of the Zevalin™ radioimmunotherapy regimen versus a standard course of rituximab immunotherapy for B-cell NHL. *Blood*

Zinzani PL, Ascani S, Piccaluga PP, Bendandi M, Pileri S and Tura S (2000) Efficacy of rituximab in hairy cell leukemia treatment. *J Clin Oncol* **18**: 3875–3877

© 2001 Cancer Research Campaign
